# Exploiting Nutritional Value of Staple Foods in the World’s Semi-Arid Areas: Risks, Benefits, Challenges and Opportunities of Sorghum

**DOI:** 10.3390/healthcare3020172

**Published:** 2015-03-30

**Authors:** Ilaria Proietti, Chiara Frazzoli, Alberto Mantovani

**Affiliations:** 1 European Commission, Joint Research Centre (JRC), Institute for Prospective Technological Studies (IPTS), Agriculture and Life Sciences in the Economy (AGRILIFE), Edificio Expo. C/Inca Garcilaso 3, 41092 Seville, Spain; 2 Food and Veterinary Toxicology Unit, Department of Veterinary Public Health and Food Safety, Istituto Superiore di Sanità, Viale Regina Elena 299, 00161 Rome, Italy; E-Mail: alberto.mantovani@iss.it; 3 External Relations Office, Istituto Superiore di Sanità, via Giano della Bella 34, 00162 Rome, Italy; E-Mail: chiara.frazzoli@iss.it

**Keywords:** underutilized food, antinutritional factors, contaminants, aminoacids, trace elements, nutrition, agriculture, Africa

## Abstract

Sorghum (*Sorghum bicolor* (L.) Moench) is a drought-resistant crop and an important food resource in terms of nutritional as well as social-economic values, especially in semi-arid environments. Cultivar selection and processing methods have been observed to impact on composition and functional and nutritional value of sorghum. Amino acid imbalance, cyanogenic glycosides, endogenous anti-nutrients, mycotoxins and toxic elements are among factors impairing its nutritional value. This paper reviews possible approaches (varieties selection, production practices, cooking processes) to improve the benefits-to-risks balance of sorghum meal, to mitigate the risk of deficiencies and/or imbalances and to improve effects on human nutrition. Opportunity for avoiding dietary diversification in high sorghum consumers is also discussed, e.g., tryptophan and niacin deficits potentially related to pellagra, or unavailability of proteins and divalent cations (e.g., Fe, Zn) due to the antinutrient activity of phytic acid and tannins. As potential candidate for production investments, the role of sorghum in preserving biological diversity is also considered.

## 1. Introduction

From the Neolithic age, technology enabled humans to raise land productivity, thus satisfying the increased demand for food. Since the 1950s, the progress in plant breeding, agricultural sciences as well in the use of inorganic fertilizers and pesticides did support a widespread exponential growth of the yields for major food crops; as a consequence, most industrial countries achieved food surpluses by the second half of the 20th century. Nevertheless, this protracted intensive exploitation brought to the depletion of most fertile soils, resulting in environmental degradation. To add further concern, by 2050 the world’s population is estimated to reach nine billion; in addition, an ever increasing portion of the world’s population will request diets with a greater content of calories and proteins. Therefore, the demand for agricultural products is expected to increase by 50% in 2030 [1] and by 80%–100% in 2050 [2]. The world’s population is forecasted to increase inhomogeneously. In Africa, for instance, population is expected to double by 2050, from one billion to about two billion. Moreover, the urban population is expected to grow faster than the rural one and the urbanization phenomenon will mostly occur in low-income countries, leading to a reduction of people engaged in agricultural activities [3]. Considering also the effects of climate change on food production (higher temperatures, more frequent and extreme weather events, leading to increase risks for flooding and drought), meeting the food demand stands out as a real challenge.

Agriculture is indeed particularly sensitive to climate change. Higher temperatures reduce yields of most food crops, while increasing weed and pest proliferation, while extreme weather events increase the likelihood of crop failures. Effects are expected to differ widely in different areas of the world; yield declines for major food crops are expected mainly in the low-income countries, and in particular in Southern Asia. Besides being overall more affected, low income countries are also expected to be less resilient: as a consequence, the food security of a considerable number of people will be put at risk.

Modern agriculture mostly relies on a small number of crops and varieties selected due to their proneness to intensification, which clearly limits its resilience in a scenario characterized by major changes and challenges [4,5,6]. In contrast, maintaining crop diversity can better preserve soil fertility and reduce the vulnerability of the agricultural system to pests and diseases [4,5,6]. Moreover, diversified diets determine better nutrition and health, with direct benefits for human productivity and livelihoods. Therefore, the food and agriculture sector needs to develop and use adaptive strategies, such as the exploitation and protection of underutilized food resources.

Traditional indigenous crop species (or landraces), e.g., millets and sorghum, which are regularly used in some low income countries or communities, represent underutilized crops (compared to maize, wheat or rice) in high income countries [5,7]. As they are farmed in difficult environments, these varieties are generally resistant to drought, pests and diseases, tolerant to abiotic impacts and represent valuable subsistence crops for many farmers around the world.

Sorghum is one of the major traditional staple food crops in many developing countries; it represents an important subsistence crop for millions of people in the semi-arid tropics of Africa, Asia and Central America. This cereal is resilient [8,9], as it has good drought and pest resistance and it is often grown in areas where it is difficult to cultivate other food grains. Sorghum is widespread, nutritious, easy to grow and well adapted to hot and arid climates thus, it represents a good crop for exploitation.

## 2. Sorghum and Nutrition Security

Sorghum is the fifth largest most important cereal in the world agricultural economy, after wheat, maize, rice and barley, and the second (after maize) in sub-Saharan Africa. In 2013, the global area cropped with sorghum was 42.3 million hectares and the worldwide production was 61.5 million metric tons; the USA, Nigeria, Mexico, India and Ethiopia are the main producers [10]. Together with millet, sorghum represents a main source of energy and protein for about one billion people in the semi-arid region of tropics and it is part of the staple diet of more than 300 million people in developing countries, representing their major source of energy and nutrients [11]. In particular, in Africa, sorghum is a basic staple food for many rural communities, especially in drought prone areas, characterized by shallow and heavy clay soils; thus, it is a subsistence food crop for many food insecure people [12].

A wide variety of traditional food products and recipes are based on sorghum. The cereal is boiled like rice, brewed for beer production, baked into flatbreads or cracked for porridge preparation. Besides providing calories, sorghum has actual nutritional value in principle, because of its content of protein, vitamins, both fat-soluble (D, E and K) and of B group (except for B12), as well as minerals, such as iron, phosphorus and zinc. In particular, a recent study classifies sorghum genotypes as source of vitamin E but highlight how the analyzed genotypes showed low contents of carotenoids [13]. In composition, sorghum grain compares favorably with some other cereals: it has a similar protein content to wheat but higher than maize and rice, while the essential amino acid composition of sorghum is comparable to maize or wheat due to the limited content of threonine, arginine and, especially, lysine [14,15]; in particular, sorghum's main storage proteins, the kafirins, are devoid of the essential amino acid lysine; thus, the abundance of kafirins in a given sorghum variety has a direct negative impact on its nutritional value. Iron content of sorghum is lower than millet but is higher than wheat, maize and rice [14,15].

As a further interesting aspect, sorghum is considered suitable for people with coeliac disease and gluten intolerance due to the lack of gluten [16,17,18]. Indeed, individuals with coeliac disease may not consume enough dietary fiber; thus, sorghum whole grains could usefully complement their diets. The impact of this aspect, although not currently assessable in developing areas, might be interesting in western populations, where the incidence of coeliac disease and gluten intolerance is an increasing phenomenon. To date, sorghum does not figure among important commodities in the North American and European food basket, but its importance as ingredient in multigrain and gluten-free cereal products is known. Sorghum might provide a good basis for gluten-free cookies and bread, thus increasing the range of alternative food products available to people suffering from coeliac disease.

## 3. Hazards to Be Identified and Managed

The nutritional value of food is increasingly recognized to be affected by intrinsic undesirable components as well as contaminants [19]. Therefore, like other traditional foods [20], the nutritional value of sorghum has to be supported by the identification and management of undesirable components. These include endogenous substances that directly interfere with nutrients as well as exogenous contaminants to which sorghum is particularly liable, such as mycotoxins and toxic trace elements up-taken from soil.

### 3.1. Endogenous Factors

#### 3.1.1. Protein Content and Aminoacid Compositions

A major issue for sorghum is the low digestibility of its grain compared with other cereals, like wheat, maize, and rice [21,22,23]. The low digestibility of sorghum is presumably due to the proteins high cross linking and their location primarily on the periphery of the protein bodies [24,25]. Among sorghum proteins, kafirins (aqueous alcohol-soluble prolamins) are the major storage protein in the kernel, comprising about 70%–80% of whole grain flour protein [26]. Kafirins have poor nutritional quality, because of the scarce content of essential amino acids (especially lysine, but also tryptophan and threonine); in addition, they are the sorghum proteins with the slowest digestibility [27].

Like other cereals, sorghum proteins are deficient in the essential amino acid lysine as well as in the sulfur-containing amino acids [14,27]. In contrast, sorghum proteins contain a relatively high proportion of leucine, in particular compared to isoleucine, which determines an unfavorable leucine/isoleucine balance. An excess of leucine can interfere with the conversion of tryptophan to niacin (vitamin B3 or nicotinic acid), hence increasing the risk of niacin deficiency which, in its turn, may result in the pellagra disease, one of the most dramatic clinical conditions related to vitamin deficiency [28,29]. Historically, pellagra is related to a maize based diet, but it has also been reported in sorghum eating populations: in Hyderabad, India [28], in South Africa, in Southern Europe in the 18th century and the USA following the civil war [29]. Still nowadays, indigent groups eating unbalanced and monotonous diets based on high consumption of sorghum and with a low protein intake should be considered as at risk of developing pellagra [28,29].

#### 3.1.2. Cyanogenic Glycosides

Most sorghum plants contain cyanogenic glycosides, although the quantity depends on several factors like the cultivar, the distribution in the plant tissues and the environment. The main cyanogenic glycoside, called dhurrin, is hydrolyzed by the β-glucosidase to produce p-hydroxybenzaylaldehyde and hydrocyanic acid or prussic acid, resulting in acute cyanide poisoning. The β-glucosidase may originate from the plant material or from certain gut microflora of exposed animals or humans [30]. Dhurrin is mostly located in the green part of the plant, namely the leaves and the stem of growing plants, while it is not present (or only at low level) in the caryopses and stalks of mature plants [31]. Therefore, Dhyrrin may represent a threat for animals feeding on sorghum materials, rather than for humans. Nevertheless, chronic effects of cyanogenic glycosides consumption are associated with long‑term intake of cyanide-containing food in individuals with poor nutritional status. Cases of cyanide poisoning are most notable in cassava-eating and, to a lesser extent, in sorghum-eating populations [32]; therefore, under certain circumstances and upon repeated and prolonged intake, sorghum meal may provide sufficient dhurrin to elicit toxicity in humans. Cyanide poisoning may have serious consequences, especially in debilitated organisms: it determines a decrease in the utilization of oxygen in the tissues, causing distress of the circulatory, respiratory, nervous and digestive systems: in some cases death may occur [33].

#### 3.1.3. Antinutrients

The nutritional profile of sorghum can be compromised to a certain extent by its content and activity of antinutrients (AN): phenolic compounds, mainly condensed tannins, and phytic acid. Both AN groups interact negatively with the bioaccessibility of essential elements in the digestive tract, in particular iron and zinc; moreover, tannins further reduce the digestibility of sorghum’ proteins. The general mechanism involves the formation of insoluble complexes at physiological pH, due to the ability of phytic acid and tannins to bind proteins and divalent cations. The tannins are also able to bind human gut enzymes involved in the cereal digestion. As a consequence, the AN elicit an unbalanced intake of essential elements as well as reduce the availability of metabolizable energy and amino acids.

Tannins are found in many different families of the higher plants and their content may be high in many foods of vegetable origin, including cereals, legumes, fruits, nuts and beverages, such as tea, wine and cocoa. As a consequence, they are an integral part of daily diet. Tannins have different biological effects in human and animal nutrition because of their ability to chelate metal ions, form complexes with macromolecules and to act as antioxidants. They are able to form complexes with numerous types of molecules, including proteins, carbohydrates and enzymes involved in their digestion, polysaccharides and bacterial cell membranes; besides substances present in foods, tannins can bind endogenous proteins, such as digestive enzymes, inhibiting their activities. As a consequence, tannins reduce the digestibility of proteins, with a subsequent increase in fecal nitrogen excretion, affect the glycaemic and insulinemic responses and increase the fecal fat excretion [34]. Tannins also affect the absorption of trace minerals by forming insoluble complexes in the gastrointestinal tract. Minerals chelated by tannins are not bioavailable for the organism, thus a diet based on consumption of large quantities of tannin-rich food, such as sorghum, is associated with minerals deficiency diseases, such as iron-deficiency anaemia [35,36].

Phytic acid (PA) is the primary storage compound of phosphorus in cereals, legumes, nuts and oil seeds: it accounts for up to 90% of total phosphorous content and contributes as much as 1.5% to the seed dry weight [37]. Its principal functions in seeds are the storage of phosphates as source of energy and the antioxidant activity for the germinating seed [38,39]. The amount of PA in plants is very variable and, presumably, it depends on growing conditions and harvesting techniques [40,41]. Nevertheless, phosphorus in PA is mostly not bioavailable to monogastric animals, including humans, due to insufficient degradation capabilities in their gastrointestinal tract under the pH conditions of the small intestine [39,41,42].

Like tannins, also PA has the ability to chelate metal cations, primarily iron, zinc, calcium, as well as proteins and digestive enzymes, such as pepsin, amylase and trypsin. The formation of insoluble complexes with metals and proteins determines their unavailability as nutrients, and can lead to deficiencies in populations where staple foods like sorghum represent the principal source of nutrition.

In Africa, sorghum is frequently used for preparing weaning food for children: the intake of phytates has a direct correlation with the poor iron and zinc status commonly seen in weaned, preschool children after 6 months of age in low-income countries, e.g., Malawi [43,44,45]. Additional studies have shown the PA ability to inhibit the absorption of further essential minerals, including calcium [46] and magnesium [47]; in particular, the PA-induced impaired bioaccessibility of zinc [48,49] may be of particular relevance for the well-being of the developing organism.

### 3.2. Exogenous Factors

Sorghum is susceptible to exogenous contaminants, such as the presence of mycotoxins upon mold infestation and the accumulation of toxic -elements from the environment.

#### 3.2.1. Mycotoxins

While acting as AN, the phenol and tannin contents meanwhile enable sorghum to be more resistant to mold infestation, diseases and damage [14,50]. Moreover, owing to sorghum’s hard seed coat, the severity of mycotoxin contamination is lower compared to maize: kernels of maize were four times more likely to be contaminated with *Aspergillus* spp than comparable samples of sorghum [51]. However, despite its relative resistance to the infestation, fungal growth and mycotoxin production constitute one of the major biotic constraints to sorghum quality and production worldwide. This may be due, at least in part, to the fact that sorghum is predominantly grown in areas of the world with underdeveloped agricultural practices, including facilities for grain storage, and harsh climatic conditions. Mycotoxins contamination in agricultural commodities has considerable impacts on health (food safety and food security) and economic and it represents a major burden, especially for developing countries. The consequences of mold contamination are massive in terms of production loss and reduced food security. Singh and Bandopadhyay [52] assessed the production losses due to sorghum grain mold from 30% to 100% depending on cultivar, time of flowering and prevailing weather conditions during flowering to harvesting; Chandrashekar *et al.* [53] estimated the annual economic losses in Asia and Africa as a result of grain mold to more than US$ 130 million. Mycotoxin contamination can be associated with microscopic molds with no gross wastage of products: however, health risks associated with mycotoxins make the contaminated commodities unsuitable for internal marketing or export. Indeed, different mycotoxins have a wide and diverse range of severe toxic effects such as carcinogenicity (e.g., aflatoxins), endocrine disruption (zearalenone), renal toxicity (e.g., ochratoin A) and neurotoxicity (e.g., fumonisins) [54]. The contamination is unpredictable and can occur during any stage of the value chain, in the field as well as during harvest, transport and storage, representing a unique challenge to food safety management. In addition, the presence of mycotoxins in feed materials, including those derived from sorghum grains, leaves and stalks, should not be overlooked: the toxicity for farm animals and in some cases the feed-to-food carry-over of residues (e.g., the M1 aflatoxin metabolite in milk) can significantly impair the production and availability of foods of animal origin [55]. Several studies reported mycotoxins contamination of sorghum, especially in tropical countries, where high temperatures, humidity and unseasonal rains during harvest can lead to the growth of a wide range mycotoxigenic fungi including *Aspergillus*, *Alternaria*, *Penicillium* and *Fusarium* spp. [50,56,57]. Mycotoxins reported in sorghum food and feed in different low-income countries, include: aflatoxins [58,59] and fumonisins [60] in India; aflatoxins and fumonisins in Brazil [61]; aflatoxins, fumonisin B1 and zearalenone in Botswana [62]; deoxynivalenol and fumonisin B1 in Cameroon [63]; deoxynivalenol, fumonisins and zearalenone in Ethiopia [64]; aflatoxins in Malawi [65]; aflatoxin B1, ocratoxin A and zearalenone in Nigeria [50]. Although sampling and analytical differences may impair the ready comparison among studies, the data overall indicate that aflatoxins and fumonisins may be the priority contaminants in most situations. Moreover, sorghum is highly vulnerable to *Alternaria* toxins, an emerging group on mycotoxins on which toxicological knowledge is still very limited [66].

#### 3.2.2. Uptake of Toxic Trace Elements

One major characteristic of sorghum grain is the ability to retain trace elements from the soil and water [67,68,69,70,71,72,73]. The concentrations of elements taken up by the cereal may be related to the stress due to deficiencies or excesses in mineral nutrients [74]. This prerogative can represent a serious issue in case of polluted soils intended for agricultural purposes, as the toxic elements can enter the food chain. Sites near industries are often contaminated with toxic metals; besides, application of low-quality fertilizers containing heavy metals, such as cadmium, increased metal content in the soils [73]. Depending on the content in soil and water, sorghum, like other grains, can show a significant uptake of arsenic [71], cadmium [70,72] and lead [70]. Nevertheless, the majority of the heavy metals are retained by the roots and a very small amount is accumulated into apical part. According to Angelova *et al*. [75], the order of the depots for accumulation in sorghum were roots > leaves > stems > grains; thus, as for cyanogenic glycosides there is a potential concern for animal feeds.

A less-known and apparently peculiar feature of sorghum is the relationship with fluorine. Fluorosis is a serious public health problem in many parts of the world where drinking water contains more than 1 mg/L of fluoride. Fluorosis affects dental health, leading to enamel defects, and—at higher intake levels—skeletal health, leading to abnormal growth and structure of the bone tissue with severe consequences to the spine, movements and joints. Drinking water represents the main source of fluoride intake, but some food commodities such as sorghum, chilies and black tea also represent significant sources [67].

In certain parts of India fluorosis is endemic; in most of these regions, sorghum is a staple food. Krishnamachari and Krishnaswamy [76] found that in Indian fluorotic areas the genu valgum, a clinical manifestation of bone deformation, was more frequent in sorghum-eating populations. In a more recent study, Indian children who consumed sorghum had a 2.67-fold higher chance of getting severe dental fluorosis compared to those who did not [77]. Moreover, Lakshmaiah and Srikantia [78] reported that retention of fluoride was significantly higher on a sorghum diet than on rice. Similarly, fluorosis is endemic also in the African Rift Valley. In Uganda Rwenyonyi *et al.* [79] found a high prevalence of dental fluorosis also in areas with low-fluoride water, indicating a plausible role of the diet. A study conducted in Western Uganda by Wandera *et al.* [80] on the fluoride content of weaning food items showed that sorghum and millet had the highest concentrations of fluoride. Checking the geochemistry of lands where sorghum is cultivated as food (or also feed) crop would support the management of the presence of toxic elements.

## 4. Challenges and Opportunities

### 4.1. Sorghum and Environmental Quality

Sorghum may be an ideal crop for the safeguarding of agricultural biodiversity, from the standpoints of both genetics and resilience. It is indeed characterized by notable genetic diversity: more than 30,000 selections in the world genetic collections exist [81]. Sorghum is mainly grown in semi-arid areas with high drought and low rainfall, that are conditions poorly favorable to the production of other grain crops; therefore, it is well suited to all Africa’s climatic zones, being able to withstand periods of water-logging [11]. Sorghum can be indeed cultivated over a wider range of ecological conditions than the majority of the food crops, as it can adapt well to both temperate and tropical zones [82].

Sorghum is a versatile crop. In industrialized countries like USA, it is mainly grown for the production of animal feed (forage or grain), in the manufacture of ethanol and as bioenergy crop for production of biomass, whereas in Africa and Asia sorghum is grown primarily for human consumption. Sorghum is a valuable crop also due to the numerous utilizations of each part of the plant within different agricultural systems. The whole plant is often used as forage, silage or hay, while the stems for building, weaving or firewood; stems of some varieties are also used as biomass for biogas production or processed for sugar and syrup. The seeds are used in the livestock sector, as feed for poultry, cattle and swine [82]. Other industrial products, like vegetable oil, adhesives, waxes or dyes are obtained by sorghum processing. Finally, due to its ability to chelate metals, sorghum has recently been proposed as a phytoextraction plant in the remediation of soil polluted by metals [70,71].

Phytoextraction is a green, cost-effective, promising technology that aims to decrease the concentration of chemical elements in contaminated soils by growing metal-accumulating plants. Several studies reported that cropping of sorghum may be a sustainable and effective technique for partial decontamination of heavy metal contaminated soils and organic pollutants due to its fast growth rate and large amount of biomass [83,84,85,86,87]. A field trial conducted by Marchiol *et al.* [83] observed the phytoextraction potential of heavy metals (As, Cd, Cr, Cu, Ni, Pb and Zn) by sorghum in a polymetallic soil. Results showed a positive potential of phytoremediation of sorghum; in particular, Zn removal reached about 2000 g ha^−1^. In another study, the potential of sorghum for phytoremediation of soil contaminated by different concentration of chromium was investigated. The results indicated that concentration of chromium after phytoremediation by sorghum decreased between 51.2%–69.5% [88]. In a recent study, sorghum has been assessed for its ability to decrease the concentration of petroleum hydrocarbons in contaminated soil in Iran. Results showed that the concentration of total petroleum hydrocarbons decreased by 64% in 90 days, 30% higher than the rates in unplanted soil [89].

### 4.2. Sorghum and Human Nutrition

The increasing pandemic of diabetes and obesity has sparked interest in identifying dietary carbohydrates as functional ingredients for controlling blood glucose and insulin levels. Some authors suggest grain sorghum as a good functional ingredient to assist in managing glucose and insulin levels in healthy individuals [90]. Certain varieties of sorghum bran were reported to affect critical biological processes that are important in diabetes and insulin resistance [91].

The nutritional profile and value of sorghum can be improved by reducing components causing nutritional concerns and/or factors increasing the liability to toxic contamination.

A number of different processing technologies have been proposed in order to increase the digestibility of sorghum proteins. Among them, extrusion [92,93], steam-flaking and reconstitution [22] malting [94,95,96,97], fermentation [97,98,99] and popping [100,101] are the most cited.

Additionally, more sophisticated technologies like the developing of novel sorghum lines have been put in place in order to improved sorghum proteins digestibility. Sorghum mutant lines with higher protein digestibility and higher lysine content have been identified [102]. These mutants are characterized by a modified protein bodies’ shape that resulted to be invaginated in shape instead of being spherical. As a result, kafirins is of easier access for digestive protease enzymes. Recent findings indicate that the protein bodies’ invagination is the result of a single-point mutation determining the signal peptide resistant to processing [103]. Similarly, also starch digestibility of sorghum can increase the overall sorghum digestibility [22,24,104]. A recent report found that a low-frequency allele type in the starch metabolic gene (pullulanase) is associated with increased digestibility, regardless of genotypic background [105].

The management of AN is currently a major priority issue to support the use of sorghum as a valuable staple food in developing countries.

The ability of tannins and phytates to chelate trace minerals is related to negative effects in monotonous diets, especially in presence of less than adequate mineral intakes; however, in other instances their activity can result in benefits. Their positive properties are partly dependent on their ability to be absorbed and metabolized in the organism [106].

Tannins may act as antioxidant scavengers of free radicals, thus contributing to the prevention of chronic pathologies such as cardiovascular diseases and cancer [34,107,108]. The antioxidant activity of tannins can be related to their favorable redox potential and their relative stability of the aryloxy radical and free or protein-complexed condensed and hydrolyzed tannins are demonstrated to be more effective in scavenging radicals than small phenolic compounds [109]. Chelation of metals copper and iron, potential initiators of hydroxyl radical production by the Fenton and Haber-Weiss reactions, is one of the ways polyphenols exert their antioxidant activity. Phenolic compounds interfere with the oxidation of molecules by donating a hydrogen atom to radicals [34]. The positive effect is nevertheless related to the concentration: at high concentrations, with high pH and in the presence of iron, tannins can exert a pro-oxidative effect in Fenton-driven systems, by initiating an auto-oxidative process [34,109,110].

Similarly, PA, the other main sorghum AN, can also have several beneficial properties. Phytate can act as an antioxidant, by binding iron ions in solution, and thereby prevent ferric irons from participating to the generation of the hydroxyl radical OH via the Fenton reaction [39,111,112]. PA is also implicated in the delay of glucose absorption [113,114] decrease of cholesterol and triglycerides in plasma [115,116], reduction of kidney stone formation [117,118], as well as reduction of the bioavailability and, therefore, toxicity of heavy metals such as lead [119] and cadmium [120]. Moreover, tests *in vitro* have also revealed possible antitumoral effects, by reducing cell proliferation in different cell lines, such as human mammary cancer cells [121], and enhancing the immune system [39]. Due to its potential favorable effects, the setting of Recommended Daily Intakes (RDI) of phytic acid has been suggested: nonetheless, these values could be quite variable among countries as well as among different population groups, depending on the age, physiological and nutritional status. In general, suggested RDI should not be exceeded: however, vulnerable groups of high sorghum-eating populations, including children under six, pregnant women and people suffering from macro- and/or micronutrient deficiencies, should be recommended to have PA intakes lower than the RDI envisaged for populations having a balanced diet [41]. For such vulnerable groups, a varied diet and/or adequate food processing could be exploited for reducing PA’s activity.

Although more attention should be devoted to the beneficial effects of tannins and phytic acid, in the real-life situations of many developing countries, the high intake of AN and the consequent impaired bioavailability of essential elements and proteins may cause serious health disorders, especially in life stages (pregnancy, early childhood) with enhanced dietary requirements. Consequently, improving the nutritional value of sorghum would improve their nutritional status of vulnerable groups of population.

Following a balanced and varied diet is pivotal also to reduce the probability of pellagra development. Because of the sorghum leucine/isoleucine unbalanced content and the consequent niacin deficiency (due to the interference with the conversion of tryptophan to niacin) it would be important for high sorghum eating populations to complement their diet with foods rich in proteins with an appropriate amino acid balance: above all meat, fish, milk and eggs; if not, groundnuts, beans or other legumes [122].

Not only a varied diet, but also conscious consumption of sorghum and good practices can be fundamental to avoid nutritional concerns. In order to reduce the intake of dhurrin, for instance, it would be important to avoid eating sorghum caryopses and stalks from immature plants, since the cyanogenic glycoside is mostly located in the green part. Preventive measures, through good practices, are the most effective approach aimed at the inhibition, or at least reduction, of mycotoxin production. Risk reduction measures are more effective if put in place at pre-harvesting stages by selecting appropriate varieties (more resistant to mold infestation) and putting into place adequate agronomic practices. Nevertheless, proper storage, transportation and marketing, in particular by maintaining low moisture levels and eliminating insect activity, are also essential to reduce the risk of mycotoxins production. Detoxification of food contaminated with mycotoxins by biological, chemical, and physical strategies have also been widely proposed [123,124,125,126,127,128]. Nonetheless, the different methods seem to be less effective and sometimes inadvisable especially because of possible losses in nutritional quality and cost and safety concerns [129].

The chemical composition of cereal grains can vary widely, depending on environment, soil, and variety [130,131,132]. A marked influence of environmental components and conditions, as well as pesticides and other chemicals, has been reported on the amount of both phenolic compounds and phytates [133,134,135,136,137], as well as protein, iron and zinc content [14,138]. Local agro-climatic conditions as well as genetic characteristics make up the many, often poorly characterized sorghum landraces (traditional variety well adapted to local agro-climatic conditions) in developing countries; these landraces may show large differences in the amount of nutrients as well as AN profile. Two studies conducted by Proietti *et al.* [139,140] on sorghum grains purchased from local markets in Lagos (Nigeria), Dakar (Senegal) and Ouagadougou (Burkina Faso) showed very different content and activity of chelating factors and nutrients, including the modulation of cellular functional markers and estimated iron bioavailability, among the selected locally grown varieties. These marked differences among traditional landraces likely result from the interplay of the environmental component, the agronomic practices and the intrinsic properties of the varieties. Thus, an adequate characterization and selection of sorghum landraces may represent an important and feasible instrument to increase the bioaccessibility and bioavailability of nutrients and minimize the content and the biological impact of anti-nutrients in sorghum. Sorghum has also high genetic variability for carotenoids, tocochromanols, and vitamin E. In a recent study, sorghum genotypes showed high variability in the profile and content of carotenoids and tocochromanols [13].

The nutritional value of sorghum can be improved through appropriate processing methods. Processing may modify sorghum chemical composition, functional and nutritional value. Sorghum tocochromanols, α-tocopherol, and vitamin E decreased after extrusion and increased after dry heat in a conventional oven. Carotenoids content in sorghum decreased after both extrusion and the dry heat in a conventional oven [13].

Processing can also ameliorate the food, by either inactivating, destroying or removing toxic components or AN without any change in the nutritive value and acceptability of the food product [141]. In order to reduce the binding property of phytates, and thus increase the bioaccessibility and bioavailability of minerals and proteins, phytates should be in the lowest amount as possible, ideally 25 mg or less per 100 g or about 0.035% of ingested food [115]. Alternatively, according to Hurrell [142], in order to improve iron absorption, the phytates:iron molar ratio (Phy/Fe) should be below 1:1 and preferably 0.4:1. However, in composite meals containing ingredients enhancing iron absorption such as ascorbic acid or animal protein, a phytates:iron molar ratio < 6:1 is proposed [143]. As mentioned previously, the impaired bioaccessibility of zinc could represent an effect of concern. Gibson [49] proposed different ranges for phytates:zinc molar ratio (Phy/Zn), based on absorption studies in humans: a ratio <5 has been associated with high bioavailability, 5 to 15 with moderate bioavailability and >15 with low zinc bioavailability corresponding to around 50%, 30% or 15% of total zinc, respectively. Two main processes are suggested to reduce the inhibitory effect of PA on mineral absorption: the mechanical removal of PA by extraction or milling, or the enzymatic degradation of PA by activation of endogenous or addition of exogenous phytases [142].

Several processing methods have been reported to significantly reduce AN concentrations prior to consumption [14,96,97,98,99]. Nevertheless, together with reducing AN, food processing can determine a reduction of nutrients as well. Milling of cereals, for instance, removes a large PA fraction, but this treatment removes at the same time the majority of the minerals and dietary fibers of the food [39]. Similarly, soaking or extracting in aqueous solutions can remove up to 75% of PA, but loss of minerals, vitamins and water-extractable proteins also occurs [142,144]. Afify *et al.* [145] reported that soaking sorghum for 20 hours in distilled water reduces phytate content by 32.4%, but also zinc by about 30% and iron reduced by between 28.16% and 40.06%. Similar results have been stated also by Lestienne *et al.* [146], who reported a loss of about 40% of iron and 30% of zinc during soaking.

Besides processing, cookery methods, such as fermentation and cooking, as used in the traditional African recipes, modulate the content of specific AN and nutrients in sorghum. Both processes are commonly used in the preparation of sorghum porridges, one of the most common ways to eat sorghum in Africa. Fermentation appears to reduce the presence of iron-binding phenolic groups and phytate; on the other hand, cooking may reduce protein digestibility [92,97,147,148]. Cooking indeed determines the formation of polymeric units linked by intermolecular disulfide bonds in kafirins, which may be the cause of the low digestibility [104].

A study conducted by Proietti *et al.* [139], on both characterized and traditional sorghum cultivars, reported that fermentation, alone and associated with cooking, significantly reduced AN content as well as increased phytase activity of sorghum grains, leading to enhancement of the estimated bioavailability of iron and zinc. On the other hand, cooking alone did not influence the concentrations of AN. The results also confirmed that trace mineral content was influenced neither by fermentation, nor by cooking, whereas it showed consistent linkage to variety. Moreover, in the same study, the modulation of cellular functional markers in human colon-derived DLD-1 cell line exposed to raw and processed samples was also analyzed. Results showed that food processing methods are able to influence biological responses in a model of gut epithelial cells *in vitro*; in particular, fermentation significantly reduced cell proteins and glutathione peroxidase (Gpx) activity, whereas cooking reduced the cellular total protein content.

Cooking process was also found to significantly increase soluble phenolic acids in Sorghum bicolor ssp. and decrease the bound ones and anthocyanins [149].

## 5. Conclusions

Sorghum is a major grain corn in the world agricultural economy and represents an important staple food for the populations of many developing countries. The cereal is part of the diet of millions of people, representing for them a major source of energy and nutrients. Sorghum is a valuable grain, due to its content of protein and micronutrients, and it is an interesting option for coeliac and gluten intolerant people because of the absence of gluten. Nevertheless, the nutritional value of sorghum as human food, as well as a feed material for food-producing animals, is impaired by the activity of endogenous and exogenous substances. The former includes an unbalanced aminoacidic composition, the presence of cyanogenic glycosides, and antinutrients, such as phenolic compounds and phytic acid. The latter includes mycotoxins and heavy metals that jeopardize the safety of the cereal.

Strategies for the improvement of sorghum’s nutritional value include the reduction of the components (or their activity) causing nutritional concerns, good practices to reduce its liability to toxic contamination by exogenous compounds, and the compensation for its nutritional deficit by following a varied diet. Most of these strategies can be easily put in place by enhancing local knowledge and practices related to sorghum landraces selection, farming practices, storage, processing, and cooking.

Sorghum is an interesting and high potential food resource; sorghum grains can represent an ideal crop for the sustainability of the agro-food system. Considering the fast pace of population growth, the effects of climate change on food production, the nutrient depletion of soils and the increasing loss of biodiversity, meeting the global food demand will be a real challenge in the near future. To be able to adequately feed the world’s growing population, the global food sector needs to boost the production or develop adaptive strategies, including the exploitation of underutilized, often neglected, food resources like sorghum. The aware exploitation and consumption of the many autochthonous landraces, which are well adapted or resistant to adverse conditions and represent an important component of biological diversity, should be highly promoted.
